# Associations of dual-energy computed tomography-derived visceral adipose tissue quality and high-risk coronary plaque in patients with metabolic syndrome

**DOI:** 10.1186/s13244-025-02186-0

**Published:** 2026-04-20

**Authors:** Yuxue Dang, Weishi Ni, Yanhua Zhen, Xinyu Fu, Wenyue Dou, Yang Hou

**Affiliations:** Department of Radiology, Shengjing Hospital of China Medical University, Shenyang, China

**Keywords:** Visceral adipose tissue, Metabolic syndrome, High-risk coronary plaques, Dual-energy computed tomography

## Abstract

**Objectives:**

To investigate the relationship between metabolic syndrome (MetS), visceral adipose tissue (VAT), and high-risk coronary plaques.

**Materials and methods:**

This study analyzed 392 hospitalized patients who underwent dual-energy computed tomography (DECT) for cardiac and abdominal imaging between May 2021 and February 2024. VAT multimodal attenuation parameters (CT_40keV_, CT_70keV_, λ_HU_, and Eff-Z) were obtained at the third lumbar vertebral level. Multivariable logistic regression was performed to assess independent associations between VAT parameters and coronary high-risk plaques. Prediction models were established to predict the presence of high-risk plaques, and their predictive efficacy was evaluated by ROC analysis.

**Results:**

MetS patients (*n* = 182) demonstrated a higher prevalence of high-risk plaques vs non-MetS (*n* = 210) (45.6% vs 25.7%, *p* < 0.001). DECT-derived VAT parameters were significantly lower in the MetS group than in the non-MetS group (*p* < 0.001). Moreover, CT_40keV_, CT_70keV_, λ_HU_, and Eff-Z were correlated with the number of MetS components and each component, after adjusting for age and sex (*p* < 0.05). CT_40keV_ was the strongest independent predictor for high-risk plaques in patients with MetS (standard partial regression coefficient Beta = −0.761, *p* < 0.001). In the predictive models for high-risk plaques, all models incorporating CT_40keV_ demonstrated improved discriminative capability compared to traditional risk assessment approaches (standalone CACS or FRS), with AUC improvements ranging from Δ0.0729 to Δ0.205 (*p* < 0.05).

**Conclusions:**

VAT was independently associated with the presence of high-risk coronary plaques in the MetS group, and DECT-derived CT_40keV_ may be a potential biomarker for high-risk plaques. The prediction model incorporating CT_40keV_ provides incremental value for predicting high-risk plaques.

**Critical relevance statement:**

This study demonstrates that visceral fat CT_40keV_ attenuation metrics derived from DECT are strongly associated with high-risk coronary plaques in MetS patients, offering a novel imaging biomarker for early cardiovascular risk stratification.

**Key Points:**

- MetS patients exhibited a significantly higher prevalence of high-risk plaques compared to non-MetS controls.
- Dual-energy CT-derived CT_40keV_ was the independent predictor for high-risk plaques in patients with MetS.
- The prediction model incorporating CT_40keV_ provides incremental value for predicting high-risk plaques.

**Graphical Abstract:**

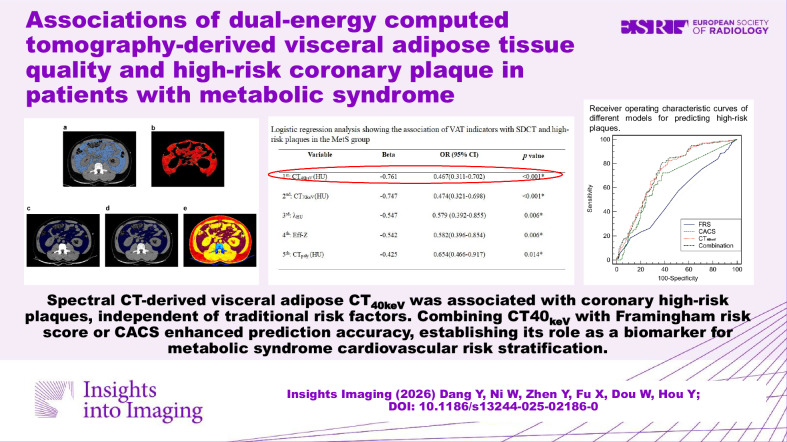

## Introduction

Metabolic syndrome (MetS) is a group of syndromes that include metabolic diseases, such as central obesity, hypertension, dyslipidemia, and abnormal blood glucose levels, and is a high-risk factor for cardiovascular diseases [1]. With the increasing prevalence MetS, there is a corresponding rise in the risk of atherosclerotic cardiovascular disease (ASCVD) and premature death [2, 3]. Current studies suggest that insulin resistance and chronic low-grade inflammation mediated by visceral adipose tissue (VAT) accumulation are key links in the occurrence and development of MetS [3–6].

Studies suggest that the adipose tissue mass itself has little effect on promoting the occurrence and development of obesity-related metabolic diseases and cardiovascular outcomes [7]; however, a series of functional disorders in adipose tissue (including adipocyte hypertrophy, immune cell infiltration, and increased expression of pro-inflammatory cytokines) play an important driving role. Therefore, it is crucial to quantify the quality (heterogeneity) of adipose tissue. Computed tomography (CT) has emerged as a robust tool for VAT quantification, enabling direct visualization and compositional profiling through attenuation values [8, 9]. Current evidence suggests that CT attenuation values reflect adipose tissue heterogeneity, correlating with lipid content, adipocyte morphology, and pathological remodeling such as fibrosis and angiogenesis [10–13]. Dual-energy CT (DECT), employing multi-energy decomposition technology, exhibits superior spatial resolution and contrast resolution, which facilitates more detailed tissue characterization in various anatomical regions [14, 15]. However, the role of DECT-derived material-specific metrics (e.g., virtual monoenergetic imaging, effective atomic number) in characterizing—VAT—phenotypic dynamics for metabolic risk stratification remains underexplored.

MetS patients exhibit heterogeneous cardiovascular risk profiles [16], highlighting the need for improved risk stratification. While high-risk coronary plaques predict adverse cardiovascular events [17], their specific association with MetS remains unclear, particularly regarding VAT characteristics. This study aimed to investigate the tripartite relationships among MetS, VAT, and high-risk plaques, and to evaluate the potential of DECT-derived multiparametric VAT quantification as a novel imaging biomarker for cardiovascular risk stratification in high-risk populations.

## Material and methods

### Study population

**Fig. 1:**
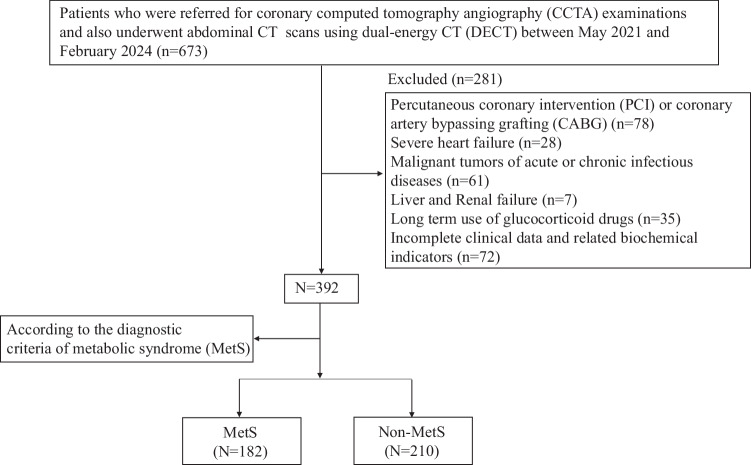
Flow diagram of patient recruitment and grouping

This was a single-center, cross-sectional observational study. Between May 2021 and February 2024, we prospectively enrolled consecutive hospitalized patients with symptoms of chest distress and pain indicative of suspected coronary artery disease (CAD) who had undergone cardiac CT and non-contrast abdominal CT (as part of routine clinical care) within a 2-week interval. Both scans were performed using a Philips Spectral IQon (Philips Healthcare). The protocol was approved by the Ethics Committee of Shengjing Hospital of China Medical University (No. 2021PS720K), and all participants provided written informed consent. The patient enrollment flow chart and study design are shown in Fig. 1. Inclusion/exclusion criteria are detailed in Supplementary Methods.

MetS was diagnosed using the 2009 internationally unified criteria [18], with waist circumference thresholds adjusted to Chinese standards [19]. Diagnosis required ≥ 3 of: (1) waist circumference, male ≥ 90 cm, female ≥ 85 cm; (2) triglycerides (TG) ≥ 1.7 mmol/L; (3) high-density lipoprotein cholesterol (HDL) levels < 1.0 mmol/L in males and < 1.3 mmol/L in females; (4) blood pressure ≥ 130/85 mmHg or antihypertensive treatment; (5) fasting blood glucose ≥ 5.6 mmol/L or diabetes treatment. Medical records were reviewed (Supplementary Methods). 10-year coronary heart disease risk was assessed using Framingham risk scoring (FRS) [20].

### Cardiac CT and coronary plaque analysis

All participants underwent coronary artery calcium score (CACS) and coronary computed tomography angiography (CCTA) following standardized protocols (Supplementary Methods).

**Fig. 2:**
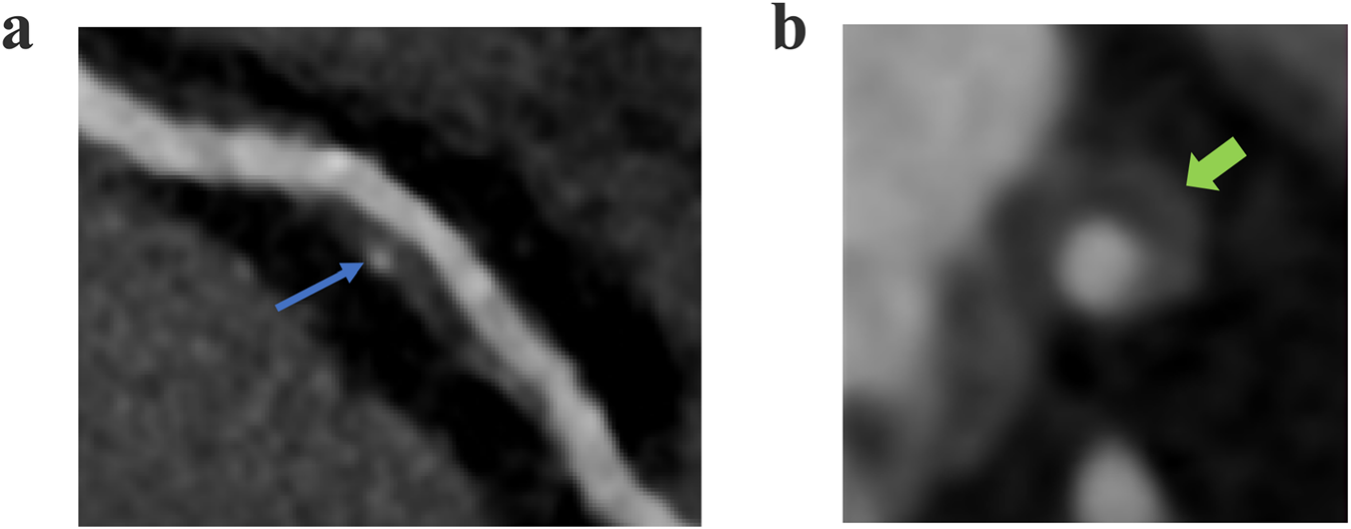
Representative examples of high-risk coronary plaques on coronary CT angiography. **a** Coronary CTA (70-year-old woman) shows a mixed plaque in the proximal left anterior descending (LAD) artery that is predominantly non-calcified; it meets three high-risk criteria—spotty calcification (thin blue arrow), low attenuation (< 30 HU), and positive remodeling. **b** Coronary CTA (67-year-old man) demonstrates a non-calcified plaque in the proximal LAD exhibiting the napkin-ring sign (thick green arrow), characterized by a central low-density core surrounded by a higher-attenuation rim adjacent to the lumen

Coronary atherosclerosis severity was categorized into non-stenotic (0% luminal stenosis), non-obstructive (1–49% luminal stenosis), or significant (≥ 50% luminal stenosis). Plaques were further classified as non-calcified, calcified, and mixed plaques according to their different components. High-risk plaques required ≥ 2 of the following CT features: (1) positive remodeling; (2) low-density plaque; (3) punctate calcification; (4) the “napkin ring” sign [21]. Figure 2 presents typical characteristics of high-risk plaques. Atherosclerotic burden was assessed using CACS and segmental involvement scores (SIS) [22, 23]. SIS, as a sensitivity assessment, assesses the degree of atherosclerosis rather than the degree of stenosis.

### VAT analysis

**Fig. 3:**
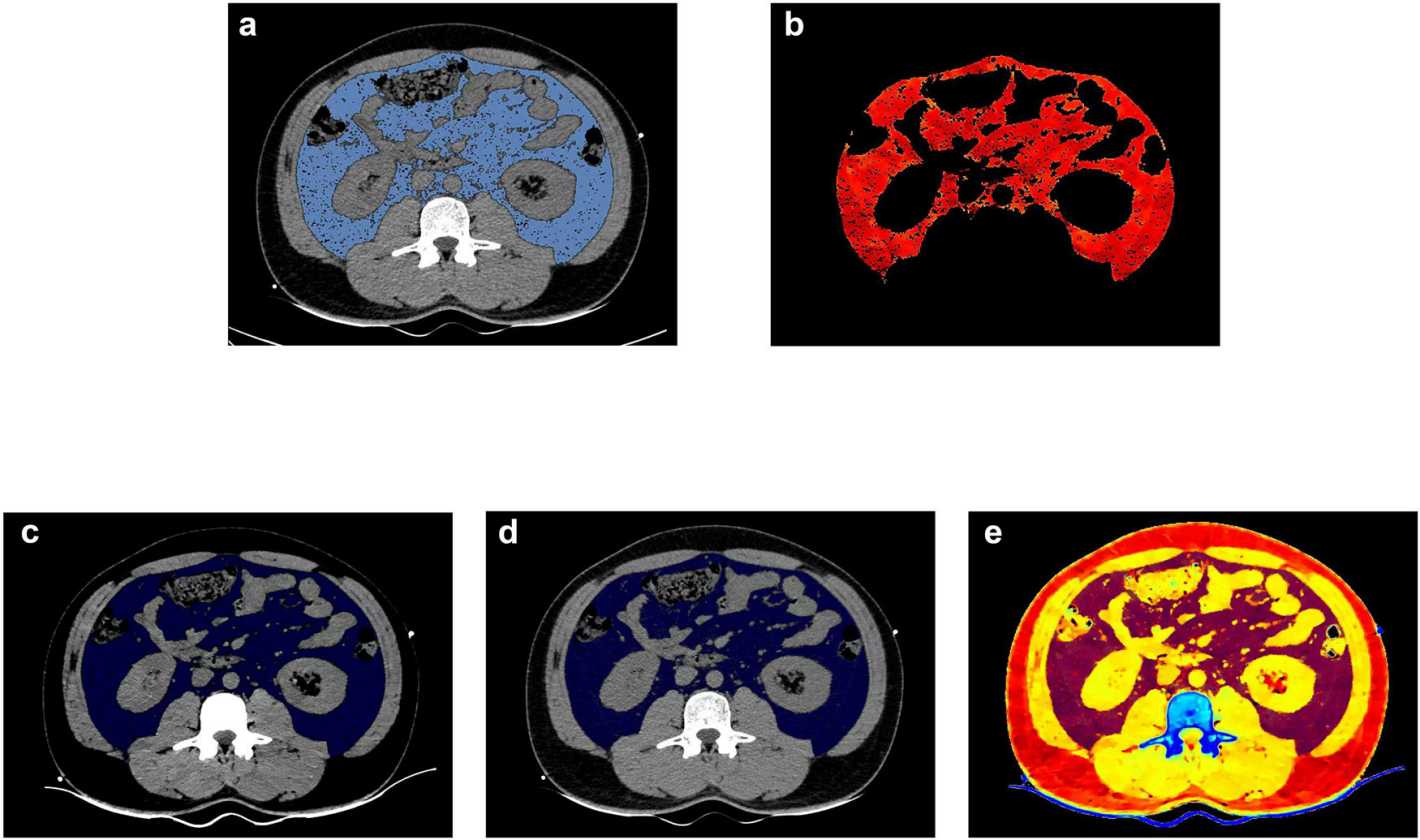
Method for measuring DECT-VAT parameters on abdominal computed tomography images at the 3rd lumbar vertebra in a case example of a 40-year-old female patient with MetS. **a**, **b** Under the conventional 120-kVp image, the visceral fat threshold was set to −190 to −30 HU, and the software automatically outlines the visceral fat tissue (**a** blue area; **b** red area), CT_poly_ = −111.3 HU, VAT = 106.5 cm^2^. **c**, **d** Virtual monochromatic imaging at 40 and 70 keV. The dark blue area is the outlined visceral fat, CT_40keV_ = −193.2 HU, CT_70keV_ = −109.8 HU. **e** The effective atomic number imaging pseudo-color map for the same case, Eff-Z = 5.77. DECT, dual-energy computed tomography; VAT, visceral adipose tissue

The unenhanced abdominal CT protocol and detailed methods for measurement of VAT are described in the Supplementary Methods. Using a dedicated workstation (IntelliSpace Portal Version 6.5, Philips Healthcare), VAT was semi-automatically segmented on a single axial section at the lumbar 3 vertebral level with a density threshold of −190 to −30 HU. The mean attenuation on conventional 120-kVp images (CT_poly_), mean attenuation on virtual mono-energetic images at 40 keV (CT_40keV_) and 70 keV (CT_70keV_), energy spectrum curve slope ((λ_HU_), and the effective atomic number image (Eff-Z) were derived from the segmented VAT region. Figure 3 presents an example of a spectral multiparametric image for measuring VAT.

### Statistical analysis

Data were analyzed using SPSS 26.0 Software (SPSS Inc.) and GraphPad Prism 8.2 software (GraphPad Software Inc.). Normally distributed data are expressed as mean ± SD (compared using *t*-test or ANOVA), while non-normal data are presented as median (upper and lower quartiles) (analyzed via Kruskal-Wallis test); categorical variables were analyzed using chi-square tests. Spearman’s correlation was used to determine the relationship between DECT-VAT parameters (CT_40keV_, CT_70keV_, λ_HU_, Eff-Z) and MetS components. For high-risk coronary plaque prediction, multivariable logistic regression incorporated (1) clinically established tools (FRS and CACS) and (2) DECT-VAT parameters identified as significant in our analysis, forming various combined predictive models. Models’ performance was evaluated using receiver operating characteristic (ROC) curves, with DeLong’s test comparing the areas under the curves (AUC). A two-sided *p* value < 0.05 was considered statistically significant.

## Results

### Participant characteristics

The study ultimately included 392 participants, 182 with MetS and 210 without MetS. The clinical baseline characteristics of the two groups are presented in Table 1. Compared to patients without MetS, those with MetS were more commonly male, had a higher body mass index (BMI) and waist circumference, and were more likely to have high blood pressure, diabetes, and a smoking history, as well as higher levels of laboratory indicators, such as TG, FPG, glycated hemoglobin A1c, and platelet-to-lymphocyte ratio (PLR), and relatively lower levels of HDL (*p* < 0.05). The rate of acute coronary syndrome during the index hospitalization in our cohort was 36.3% in the MetS group versus 25.2% in non-MetS patients (*p* < 0.05). Individuals with MetS exhibited a notably higher FRS compared to those without (*p* < 0.001).

**Table 1:** Baseline characteristics

	MetS group (*n* = 182)	Non-MetS group (*n* = 210)	All (*n* = 392)	*p* value
Clinical data				
Age (years)	60.0 (52.0–66.3)	62.0 (54.0–68.0)	61.0 (53.0–67.0)	0.268
Male (%)	87 (47.8)	74 (35.2)	161 (41.1)	0.012^*^
BMI (kg/m^2^)	26.9 (24.7–28.7)	23.4 (21.5–24.5)	24.4 (22.7–27.3)	< 0.001^*^
Waist circumference (cm)	93.0 (85.0–101.0)	80.0 (76.0–86.0)	85.0 (78.0–95.0)	< 0.001^*^
Male	96.0 (89.0–103.0)	82.0 (76.0–88.0)	89.5 (80.3–98.0)	< 0.001^*^
Female	90.0 (82.0–96.0)	79.0 (76.0–85.0)	83.0 (77.0–92.0)	< 0.001^*^
History				
Hypertension, *n* (%)	136 (74.7)	76 (36.2)	212 (54.1)	< 0.001^*^
Diabetes mellitus, *n* (%)	114 (62.6)	54 (25.7)	168 (42.9)	< 0.001^*^
Smoking, *n* (%)	49 (26.9)	45 (21.4)	94 (24.0)	0.204
Drinking, *n* (%)	42 (23.1)	31 (14.8)	73 (18.6)	0.035^*^
Prior CAD, *n* (%)	10 (5.5)	9 (4.3)	19 (4.8)	0.578
Prior MI, *n* (%)	3 (1.7)	1 (0.5)	4 (1.0)	0.249
Laboratory index				
TC (mmol/L)	4.8 ± 1.2	4.7 (4.0–5.3)	4.7 (4.0–5.5)	0.555
TG (mmol/L)	2.0 (1.5–2.8)	1.1 (0.8–1.4)	1.4 (1.0–2.1)	< 0.001^*^
HDL (mmol/L)	1.0 (0.8–1.1)	1.4 (1.1–1.6)	1.1 (1.0–1.4)	< 0.001^*^
LDL (mmol/L)	3.0 ± 1.2	2.8 (2.2–3.4)	2.9 (2.2–3.6)	0.455
FPG (mmol/L)	6.3 (5.5–7.7)	5.4 (5.0–6.1)	5.7 (5.2–7.2)	< 0.001^*^
HbA1c (mmol/L)	6.6 (5.8–7.7)	5.8 (5.3–6.6)	6.0 (5.4–7.1)	< 0.001^*^
NLR	2.1 (1.5–2.8)	2.0 (1.6–3.0)	2.1 (1.5–2.9)	0.654
PLR	114.8 (88.5–152.8)	131.7 (97.2–171.3)	122.6 (93.6–165.7)	0.004^*^
Diagnosis of ACS	66 (36.3%)	53 (25.2%)	119 (30.4%)	0.018^*^
UA	63 (34.6%)	50 (23.8%)	113 (28.8%)	0.018^*^
NSTEMI	2 (1.1%)	2 (0.95%)	4 (1.0%)	0.886
STEMI	1 (0.55%)	1 (0.48%)	2 (0.51%)	0.919
FRS	15.0 (12.0–17.0)	13.0 (10.8–15.0)	14.0 (11.0–16.0)	< 0.001^*^

*MetS* metabolic syndrome, *BMI* body mass index, *CAD* coronary artery disease, *MI* myocardial infarction, *TC* total cholesterol, *TG* triglycerides, *HDL* high-density lipoprotein, *LDL* low-density lipoprotein, *FBG* fasting blood glucose, *HbA1c* glycated hemoglobin A1c, *NLR* neutrophil-to-lymphocyte ratio, *PLR* platelet-to-lymphocyte ratio, *ACS* acute coronary syndrome, *UA* unstable angina, *NSTEMI* non-ST-elevation myocardial infarction, *STEMI* ST-elevation myocardial infarction, *FRS* Framingham risk score

^*^ Indicates *p* < 0.05

### Coronary plaque characteristics and VAT attenuation indices analysis

In the MetS group, 145 (79.7%) patients had coronary stenosis, including 86 (47.3%) with non-obstructive stenosis and 59 (32.4%) with significant stenosis. Among the non-MetS group, 134 (63.8%) had coronary stenosis, including 79 (37.6%) with non-obstructive stenosis and 55 (26.2%) with significant stenosis. Only the presence or absence of coronary stenosis was significantly different between the two groups (*p* = 0.001). In addition, the incidence of high-risk plaques was significantly higher in the MetS group than in the non-MetS group (83 patients [45.6%] VS 54 patients [25.7%], *p* < 0.001) (Table 2). In patients with high-risk plaques, the number of plaque features did not differ significantly between the two groups (*p* > 0.05). We also quantified coronary atherosclerotic load by CACS and SIS and found that CACS and SIS were significantly higher in the MetS group than in the non-MetS group (*p* < 0.01).

**Table 2:** Differences in plaque characteristics and DECT-VAT parameters between the groups

	MetS group (*n* = 182)	Non-MetS group (*n* = 210)	*p* value
Stenosis severity			
No stenosis	37 (20.3%)	76 (36.2%)	0.001^*^
Non-obstructive stenosis	86 (47.3%)	79 (37.6%)	0.054
Significant stenosis	59 (32.4%)	55 (26.2%)	0.176
Coronary plaque			
Calcified plaque	104 (57.1%)	91 (43.3%)	0.006^*^
Noncalcified plaque	85 (46.7%)	71 (33.8%)	0.009^*^
Mixed plaque	65 (35.7%)	47 (22.4%)	0.004^*^
Presence of high-risk plaque	83 (45.6%)	54 (25.7%)	< 0.001^*^
Numbers of high-risk plaque features			
2	51 (61.4%)	33 (61.1%)	0.969
3	25 (30.1%)	15 (27.8%)	0.768
4	7 (8.4%)	6 (10.9%)	0.626
CACS	16.9 (0.0–155.3)	0.0 (0.0–83.6)	0.002^*^
0	68 (37.4%)	117 (55.7%)	< 0.001^*^
1–100	55 (30.2%)	43 (20.5%)	0.026^*^
101–300	28 (15.4%)	20 (9.5%)	0.077
> 300	31 (17.0%)	30 (14.3%)	0.454
SIS	2.0 (1.0–5.0)	1.0 (0.0–3.0)	< 0.001^*^
SIS ≥ 5	53 (29.1%)	37 (17.6%)	0.007^*^
VAT indices on DECT			
CT_poly_ (HU)	−108.7 (−110.2, −106.6)	−107.0 (−109.2, −103.6)	< 0.001^*^
CT_40keV_ (HU)	−189.6 (−192.6, −185.7)	−184.5 (−189.5, −177.2)	< 0.001^*^
CT_70keV_ (HU)	−106.8 (−108.6, −105.0)	−104.5 (−107.1, −101.1)	< 0.001^*^
λ_HU_	−2.75 (−2.83, −2.68)	−2.66 (−2.76, −2.54)	< 0.001^*^
Eff-Z	5.79 (5.75, 5.86)	5.87 (5.80, 5.96)	< 0.001^*^
VAT area (cm^2^)	100.4 ± 31.5	57.8 (41.3, 85.2)	< 0.001^*^

*DECT* dual-energy computed tomography, *VAT* visceral adipose tissue, *MetS* metabolic syndrome, *CACS* coronary artery calcium score, *SIS* segment involvement score

^*^ Indicates *p* < 0.05

We compared the DECT-VAT parameters between the two groups and found statistically significant differences in these measures between the MetS and non-MetS groups (*p* < 0.001).

### Relationship between DECT-VAT parameters and MetS

**Fig. 4:**
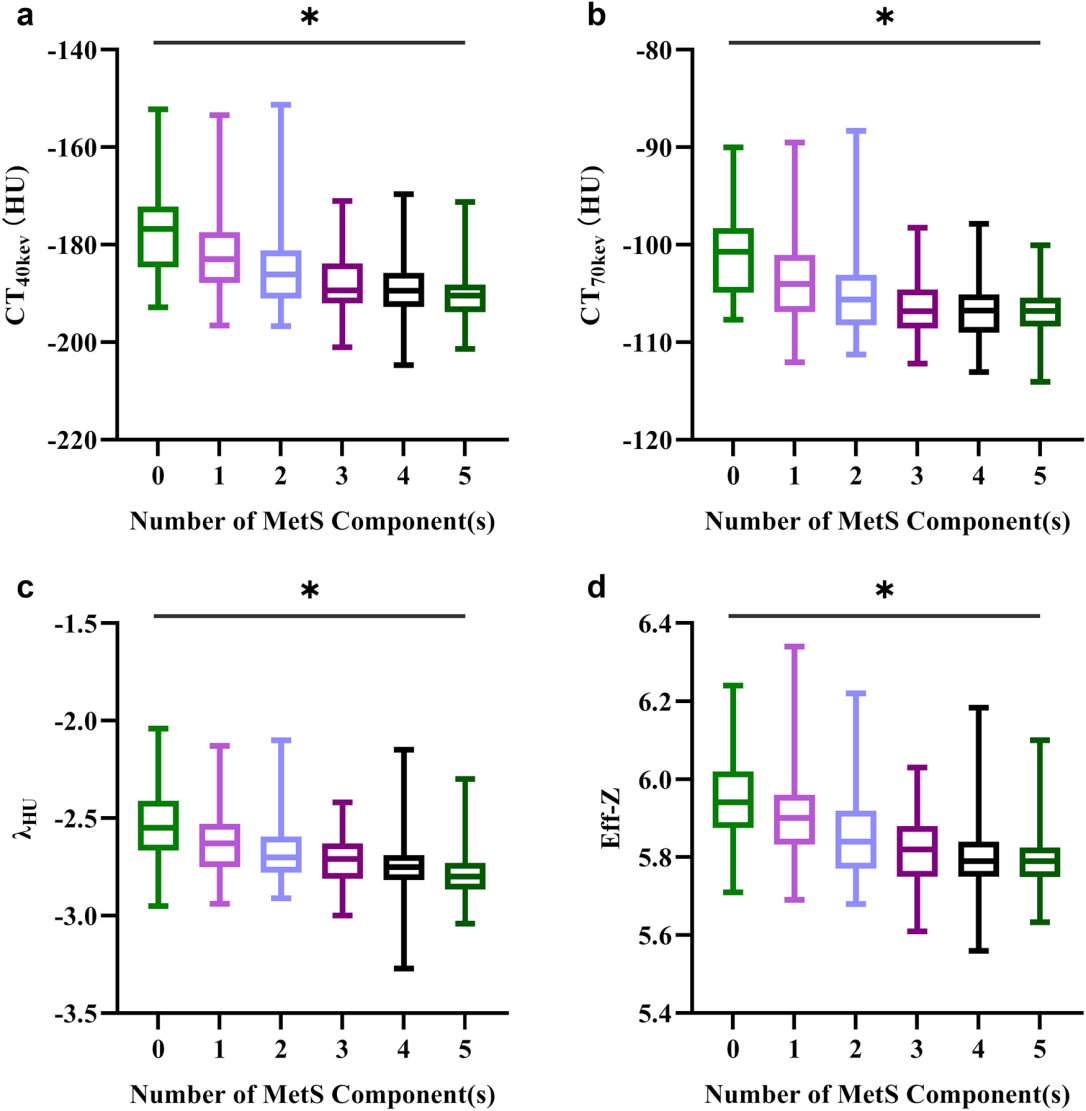
Graph showing the change in DECT-VAT parameters according to the number of MetS components; *n* = 33, 68, 109, 77, 72, and 33 for 0, 1, 2, 3, 4, and 5 MetS components, respectively. Data are presented as the median (interquartile range). ^*^p < 0.05. DECT, dual-energy computed tomography; VAT, visceral adipose tissue; MetS, metabolic syndrome

According to the MetS diagnostic criteria, subjects were divided into six groups (0–5) according to the number of MetS characteristics they had. Figure 4 shows that CT_40keV_, CT_70keV_, λ_HU_, and Eff-Z showed a downward trend as a whole with the increase in the number of MetS components, and the differences were statistically significant. Spearman’s correlation analysis showed that after adjusting for sex and age, CT_40keV_, CT_70keV_, λ_HU_, and Eff-Z were correlated with both the number of MetS and each MetS component (Table 3).

**Table 3:** Correlation between VAT indicators on DECT and MetS

Variable	*r*	*p* value	*r*'	*p* value
CT_40keV_				
Number of MetS components	−0.416	< 0.001^*^	−0.411	< 0.001^*^
Abdominal obesity	−0.404	< 0.001^*^	−0.359	< 0.001^*^
Hypertension	−0.103	0.042^*^	−0.118	0.020^*^
Hyperglycemia	−0.155	0.002^*^	−0.170	0.001^*^
TG (mmol/L)	−0.413	< 0.001^*^	−0.214	< 0.001^*^
HDL (mmol/L)	0.310	< 0.001^*^	0.259	< 0.001^*^
NLR	0.136	0.007^*^	0.153	0.002^*^
PLR	0.164	0.001^*^	0.117	0.021^*^
CT_70keV_				
Number of MetS components	−0.380	< 0.001^*^	−0.391	< 0.001^*^
Abdominal obesity	−0.379	< 0.001^*^	−0.332	< 0.001^*^
Hypertension	−0.095	0.061	−0.115	0.024^*^
Hyperglycemia	−0.117	0.021^*^	−0.139	0.006^*^
TG (mmol/L)	−0.411	< 0.001^*^	−0.208	< 0.001^*^
HDL (mmol/L)	0.256	< 0.001^*^	0.260	< 0.001^*^
NLR	0.128	0.011^*^	0.148	0.003^*^
PLR	0.145	0.004^*^	0.118	0.020^*^
λ_HU_				
Number of MetS components	−0.391	< 0.001^*^	−0.374	< 0.001^*^
Abdominal obesity	−0.382	< 0.001^*^	−0.334	< 0.001^*^
Hypertension	−0.085	0.092	−0.105	0.038^*^
Hyperglycemia	−0.169	0.001^*^	−0.174	0.001^*^
TG (mmol/L)	−0.361	< 0.001^*^	−0.191	< 0.001^*^
HDL (mmol/L)	0.306	< 0.001^*^	0.224	< 0.001^*^
NLR	0.116	0.021^*^	0.138	0.006^*^
PLR	0.151	0.003^*^	0.101	0.047^*^
Eff-Z				
Number of MetS components	−0.420	< 0.001^*^	−0.403	< 0.001^*^
Abdominal obesity	−0.425	< 0.001^*^	−0.381	< 0.001^*^
Hypertension	−0.116	0.022^*^	−0.136	0.007^*^
Hyperglycemia	−0.146	0.004^*^	−0.144	0.004^*^
TG (mmol/L)	−0.406	< 0.001^*^	−0.220	< 0.001^*^
HDL (mmol/L)	0.313	< 0.001^*^	0.249	< 0.001^*^
NLR	0.164	0.001^*^	0.206	< 0.001^*^
PLR	0.182	< 0.001^*^	0.165	0.001^*^

*r*’, adjusted for sex, age

*VAT* visceral adipose tissue, *DECT* dual-energy computed tomography, *MetS* metabolic syndrome, *TG* triglycerides, *HDL* high-density lipoprotein, *NLR* neutrophil-to-lymphocyte ratio, *PLR* platelet-to-lymphocyte ratio

^*^ Indicates *p* < 0.05

### Association of DECT-VAT parameters with high-risk plaques in MetS

We performed multivariable logistic regression to determine whether the association between DECT-VAT parameters and high-risk plaques in the MetS population was independent of traditional cardiovascular risk factors. As shown in Table 4, after adjusting for age, sex, BMI, and traditional cardiovascular risk factors (diabetes, hypertension, smoking history, drinking history, total cholesterol, TG, HDL, and low-density lipoprotein), VAT indicators on DECT were identified as significant predictors of the development of high-risk plaques in the MetS population. Due to the collinearity of the above parameters (CT_40keV,_ CT_70keV_, λ_HU_, Eff-Z, CT_poly_), we substituted the above parameters into the regression equation for analysis. By comparing the partial regression coefficients, we found that CT_40keV_ was the most powerful independent predictor (standard partial regression coefficient Beta = −0.761, OR [95% CI] = 0.467 [0.311–0.702], *p* < 0.001). We further evaluated whether CT_40keV_ was associated with the presence of high-risk plaques in different models and found that CT_40keV_ was still associated with high-risk plaques, independent of visceral fat area, stenosis severity, CACS, and SIS (complete model parameters are provided in Supplementary Table S1).

**Table 4:** Logistic regression analysis showing the association of VAT indicators with DECT and high-risk plaques in the MetS group (*n* = 182).

Variable	Beta	OR (95% CI)	*p* value
1st: CT_40keV_ (HU)	−0.761	0.467 (0.311–0.702)	< 0.001^*^
2nd: CT_70keV_ (HU)	−0.747	0.474 (0.321–0.698)	< 0.001^*^
3rd: λ_HU_	−0.547	0.579 (0.392–0.855)	0.006^*^
4th: Eff-Z	−0.542	0.582 (0.396–0.854)	0.006^*^
5th: CT_poly_ (HU)	−0.425	0.654 (0.466–0.917)	0.014^*^

*VAT* visceral adipose tissue, *DECT* dual-energy computed tomography, *MetS* metabolic syndrome,

*OR* odds ratio, *CI* confidence interval

^*^ Indicates *p* < 0.05

### Prediction models for high-risk plaques

**Fig. 5:**
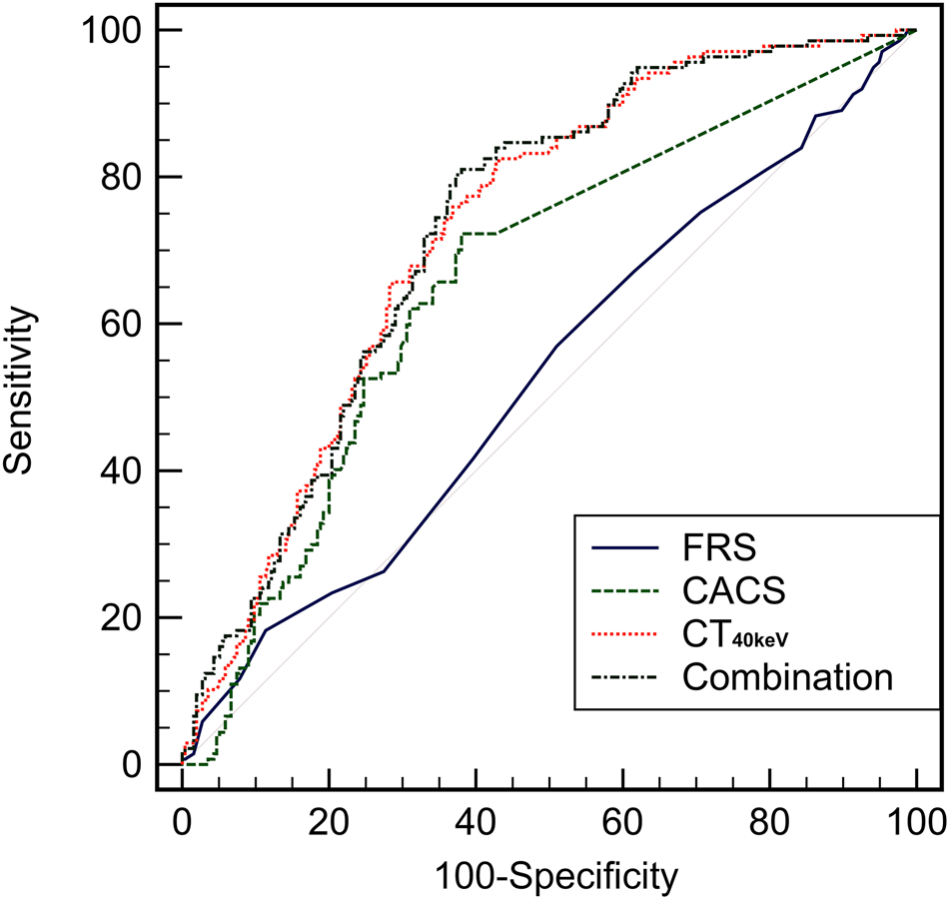
Receiver operating characteristic curves of different models for predicting high-risk plaques. FRS, Framingham risk score; CACS, coronary artery calcium score; Combination, the prediction model combining CT_40keV_ and FRS and CACS

Six models were established for the ROC analysis: Model A, FRS; Model B, CACS; Model C, CT_40keV_; Model D, FRS + CT_40keV_; Model E, CACS + CT_40keV_; and Model F, FRS + CACS + CT_40keV_. Table 5 and Fig. 5 present the ROC AUC values of different models for predicting high-risk plaques. Models D, E, and F, which included CT_40keV_, exhibited a higher AUC than the FRS or CACS alone. However, combining CT_40keV_ and FRS or CACS did not improve the performance of CT_40keV_ alone in terms of AUC.

**Table 5:** Receiver operating characteristic AUC for predicting high-risk plaques

	AUC	ΔAUC	*p* value	Sensitivity (%)	Specificity (%)
Model A: FRS	0.529 (0.478–0.579)	-	-	18.25	88.63
Model B: CACS	0.659 (0.610–0.706)	0.130 vs Model A	0.0002^*^	72.26	61.96
Model C: CT_40keV_	0.728 (0.681–0.772)	0.200 vs Model A	< 0.0001^*^	82.48	56.86
		0.0695 vs Model B	0.0793		
Model D: FRS + CT_40keV_	0.728 (0.681–0.771)	0.200 vs Model A	< 0.0001^*^	83.21	58.43
		0.0692 vs Model B	0.0753		
		0.000315 vs Model C	0.9403		
Model E: CACS + CT_40keV_	0.733 (0.687–0.777)	0.205 vs. Model A	< 0.0001^*^	82.48	59.61
		0.0746 vs. Model B	0.0454^*^		
		0.00510 vs. Model C	0.3617		
		0.00541 vs. Model D	0.4038		
Model F: FRS + CACS + CT_40keV_	0.732 (0.685–0.775)	0.203 vs. Model A	< 0.0001^*^	80.29	62.75
		0.0729 vs. Model B	0.0481^*^		
		0.00339 vs. Model C	0.6065		
		0.00371 vs. Model D	0.4872		
		0.00170 vs. Model E	0.5977		

Data in parentheses are 95% confidence intervals

*AUC* area under the curve, *ΔAUC* the difference of the area under the receiver operating curve between the two models, *FRS* Framingham risk score, *CACS* coronary artery calcium score

^*^ Indicates *p* < 0.05

## Discussion

This study investigated the relationship between visceral fat, MetS, and high-risk coronary artery plaque using DECT. The MetS group demonstrated significantly lower CT_40keV_, CT_70keV_, λ_HU_, and Eff-Z compared to non-MetS controls (*p* < 0.001). These parameters were correlated with the number and individual components of MetS (*p* < 0.05), suggesting DECT-based VAT attenuation metrics could provide a quantitative approach for characterizing visceral adiposity features associated with metabolic dysregulation. MetS patients showed a higher prevalence of high-risk plaques. Notably, CT_40keV_ was the strongest independent predictor of the presence of high-risk plaques, independent of traditional cardiovascular risk factors, coronary stenosis severity, CACS, and SIS.

Previous studies have found that MetS increases cardiovascular risk, though mechanisms remain unclear. VAT accumulation is known to be a potential cause of MetS, and VAT, as a special endocrine organ, is more strongly associated with cardiometabolic risk factors than other ectopic fat depots [8, 24, 25]. VAT is characterized by a greater ability to store and mobilize TG, higher lipolysis activity, and increased delivery of free fatty acids to the liver, ultimately leading to insulin depletion [26]. In addition, as an endocrine organ, VAT can secrete several adipokines. Compared to SAT, VAT is more prone to immune cell infiltration and the production of inflammatory cytokines, thereby causing chronic low-grade inflammation [27]. However, low-grade chronic inflammation and insulin resistance in adipose tissue can cause endothelial dysfunction, destruction of the stability of blood vessel walls, and further promote plaque instability [6, 28]. This metabolic heterogeneity of VAT, along with other systemic disturbances in MetS, may contribute to both increased coronary atherosclerotic burden (manifested by higher CACS and SIS) and a higher prevalence of high-risk plaques in these patients, thereby elevating the risk of future adverse cardiovascular events.

This study is the first to explore the feasibility of evaluating VAT attenuation by DECT. The results showed that patients with MetS had a larger VAT area than non-MetS patients, which indicates that patients with MetS had more visceral fat mass. Excessive visceral fat accumulation induces adipocyte hypertrophy, impairing their lipid storage capacity. This dysfunction drives ectopic lipid deposition in non-adipose organs, resulting in lipotoxicity that disrupts glucose metabolism, exacerbates systemic insulin resistance, and triggers chronic inflammation—ultimately promoting cardiometabolic disorders and related comorbidities [29–31]. DECT-VAT attenuation indicators can be used to assess lipid content and thus reflect functional changes in adipose tissue. In addition, after adjusting for sex and age, VAT indicators were associated with all MetS components (*p* < 0.05). At the same time, with the increase in MetS components, the DECT-VAT attenuation multimodal indices showed a gradual decreasing trend. Earlier studies [10] in the Framingham Heart Study cohort explored the correlation between CT attenuation of VAT and adverse cardiometabolic risk factors using conventional CT, suggesting that lower CT attenuation values were associated with high blood pressure, insulin resistance, TG levels, and low HDL. The DECT-VAT attenuation indicators can be used as a direct method to evaluate the quality of visceral fat to determine its relationship with obesity-related metabolic diseases. Besides, our data revealed correlations between the VAT index and the inflammation biomarkers neutrophil-to-lymphocyte ratio (NLR) and PLR, which are readily available in clinical labs and are associated with cardiovascular risks and adverse cardiac outcomes [32, 33]. Our research found that the PLR level in the MetS group was relatively high, and the difference was statistically significant, suggesting that higher inflammation and oxidative stress levels in patients with MetS provide a suitable pathophysiological environment for high-risk plaque development. While high-risk plaques are transient, MetS-driven chronic inflammation (via visceral adipose-derived cytokines, insulin resistance, and dyslipidemia) fuels new unstable plaques, sustaining dynamic vulnerability [6, 27, 28]. Critically, MetS-associated metabolic disturbances (e.g., dyslipidemia, oxidative stress, and chronic low-grade inflammation) directly link visceral adiposity to cardiovascular risk beyond traditional cardiovascular risk factors. Here, DECT-VAT indicators may serve as novel imaging biomarkers, enabling quantitative assessment of how visceral fat dysfunction drives plaque destabilization—thereby offering mechanistic insights into MetS-driven coronary vulnerability.

Given the increasing incidence of MetS worldwide and its associated ASCVD risk, early identification of high-risk populations is critical. Our study showed that DECT-VAT attenuation indicators, particularly CT_40keV_, correlated significantly with the presence of high-risk plaques, independent of traditional risk factors, visceral fat area, stenosis severity, CACS, and SIS (*p* < 0.001). As an increasingly widely used dual-energy CT technology, DECT overcomes the shortcomings of traditional CT with a single parameter and can obtain both low- and high-energy data in a single scan; moreover, DECT can provide spectral and quantitative virtual single-energy image results for various energy levels, including λ_HU_ and Eff-Z, and realize comprehensive evaluation of tissue characterization [34, 35]. In unenhanced DECT, virtual single-energy images at 40 keV can significantly enhance X-ray attenuation differences in lipid-rich structures [36]. This sensitivity to lipid content enables CT_40keV_ to detect subtle variations in visceral fat quality, reflecting the changes in the metabolic status of the body. Consequently, CT_40keV_ correlated significantly with MetS components and high-risk plaques. These findings suggest that abnormal VAT functionality (beyond mere fat mass accumulation) contributes to cardiometabolic disease, potentially establishing CT_40keV_ as a novel biomarker for cardiovascular risk assessment in MetS. Although adverse metabolic factors in patients with MetS promote uneven plaque composition with high-risk plaque characteristics and increased lipid components [37, 38], these characteristics are more strongly associated with poor cardiovascular outcomes [39–41]. Traditional MetS criteria fail to assess plaque vulnerability. Future studies are needed to determine the predictive value of DECT-VAT parameters (especially CT_40keV_) for the metabolic activity of visceral fat. If confirmed, their incorporation into risk assessment systems would have significant clinical implications.

We observed no significant difference in non-obstructive stenosis, significant stenosis or CACS (> 100) between MetS and non-MetS groups, despite higher high-risk plaque prevalence in MetS—a plausible paradox. This discrepancy reflects distinct pathophysiological processes: (i) stenosis severity quantifies overall luminal narrowing from total (both calcified and non-calcified) plaque burden, (ii) CACS denotes chronic calcified plaque load, indicating stabilized, low-inflammatory phase [42, 43]; while (iii) high-risk plaques (e.g., lipid-rich cores with positive remodeling) represent early, inflammation-driven vulnerability, primarily mediated by MetS-associated metabolic inflammation prior to significant calcification or stenosis. PROSPECT II showed future culprit lesions mostly arise from non-obstructive segments [44], with residual risk remaining even when CACS is zero, chiefly from non-calcified plaques [45, 46]. Thus, the absence of group differences in stenosis or high CACS does not contradict the higher high-risk plaque prevalence; it underscores that MetS drives early inflammatory plaque instability—poorly captured by anatomical metrics.

While CACS correlates with the severity of coronary atherosclerosis and future cardiovascular risk [47], its inability to assess non-calcified plaques limits its standalone predictive value. The FRS remains widely used for cardiovascular risk prediction and clinical decision-making, but it often classifies patients into indeterminate-risk groups, necessitating supplemental biomarkers for improved stratification [48]. In this study, the CT_40keV_-enhanced prediction model demonstrated significantly better discriminative performance than FRS or CACS alone (ΔAUC: 0.0729–Δ0.205; *p* < 0.05; Table 5). The clinical implications are twofold: (1) DECT-enabled VAT characterization could optimize risk stratification during routine abdominal CT examinations without additional radiation exposure, and (2) CT_40keV_ may identify high-risk individuals warranting intensive lifestyle interventions. While these results are promising, external validation in multicenter cohorts is warranted to establish generalizability.

This study has several limitations. First, this was a single-center, cross-sectional study without longitudinal follow-up data to assess associations with future cardiovascular events. Second, our study focused on acutely ill in-patients; among them, 36.3% of MetS and 25.2% of non-MetS participants presented with acute coronary syndrome. The stress-induced cytokine surge during acute illness may transiently alter VAT attenuation through inflammatory mechanisms distinct from chronic metabolic changes in stable MetS. These findings should therefore be interpreted cautiously regarding stable outpatients. Even so, VAT attenuation derived from routine abdominal DECT may help identify individuals at elevated cardiovascular risk, particularly in surgical candidates for whom preoperative stratification could be considered. Third, key metabolic variables—including dyslipidemia severity, lipid-lowering treatment effects, and chronicity of hypertension or diabetes—were not systematically accounted for, potentially influencing observed plaque characteristics. Additionally, the use of Chinese-specific MetS diagnostic criteria (e.g., waist circumference thresholds) may affect the generalizability of our findings to other ethnic populations with differing metabolic risk profiles. Finally, further mechanistic studies at the cellular and histological levels are needed to confirm the feasibility of CT_40keV_ for capturing potential visceral fat features.

In conclusion, this pioneering DECT study identifies CT_40keV_ as a novel biomarker for high-risk coronary plaques in MetS patients, independent of traditional risk stratification tools. The integration of CT_40keV_ with conventional models (FRS/CACS) significantly enhances plaque vulnerability assessment.

## Supplementary information

ELECTRONIC SUPPLEMENTARY MATERIAL

## Data Availability

The datasets used and/or analyzed during the current study are available from the corresponding author on reasonable request.

## Abbreviations

ASCVD: Atherosclerotic cardiovascular disease
AUC: Area under curve
BMI: Body mass index
CACS: Coronary artery calcium score
CAD: Coronary artery disease
CCTA: Coronary computed tomography angiography
CT_40keV_: Mean attenuation on virtual mono-energetic images at 40 keV
CT_70keV_: Mean attenuation on virtual mono-energetic images at 70 keV
CT_poly_: Mean attenuation on conventional 120-kVp images
DECT: Dual-energy computed tomography
Eff-Z: Effective atomic number image
FRS: Framingham risk score
HDL: High-density lipoprotein
MetS: Metabolic syndrome
NLR: Neutrophil-to-lymphocyte ratio
PLR: Platelet-to-lymphocyte ratio
ROC: Receiver operating characteristic curves
SIS: Segmental involvement scores
TG: Triglycerides
VAT: Visceral adipose tissue
λ_HU_: Energy spectrum curve slope
