# Supplementary material for: Associations of dual-energy computed tomography-derived visceral adipose tissue quality and high-risk coronary plaque in patients with metabolic syndrome
Source: Insights Imaging. 2026 Apr 20;17:108. doi: 10.1186/s13244-025-02186-0 (PMC13096456; doi:10.1186/s13244-025-02186-0)
Supplement: Supplementary file 1 — ELECTRONIC SUPPLEMENTARY MATERIAL [file 13244_2025_2186_MOESM1_ESM.docx]

**Supplemental Methods.**

**Study population**

Between May 2021 and February 2024, we prospectively enrolled consecutive in-patients who (i) underwent coronary CTA for clinically suspected CAD—typical or atypical chest pain, exertional dyspnoea, or equivalent symptoms as determined by the attending cardiologist—and (ii) had non-contrast abdominal CT performed within 2 weeks of the cardiac CT for routine clinical reasons (e.g., evaluation of abdominal pain, infection screening, pre-operative assessment, or follow-up of known intra-abdominal pathology).

The inclusion criteria were as follows: (1) age ≥18 years old, and (2) BMI ≥18.5 kg/m^2^. The exclusion criteria were as follows: (1) prior percutaneous coronary intervention or coronary artery bypass grafting; (2) severe heart failure; (3) combined with malignant tumors or acute and chronic infectious diseases; (4) liver and kidney failure; (5) long-term use of glucocorticoid drugs; and (6) incomplete clinical data and related biochemical indicators.

Clinical baseline data, disease history (hypertension, diabetes, alcohol, smoking history), and laboratory indicators (total cholesterol [TC], triglycerides [TG], high-density lipoprotein [HDL], low-density lipoprotein [LDL], fasting blood glucose [FBG], glycated hemoglobin A1c [HbA1c], neutrophil-to-lymphocyte ratio [NLR], platelet-to-lymphocyte ratio [PLR]) were collected. Acute coronary syndrome (ACS)—comprising unstable angina, non-ST-elevation myocardial infarction, and ST-elevation myocardial infarction—was diagnosed in accordance with the Fourth Universal Definition of Myocardial Infarction (2018), as endorsed by the European Society of Cardiology and the American College of Cardiology/American Heart Association [1]. Framingham risk score (FRS) data were calculated using the established categorical model using the following variables: age, sex, systolic blood pressure, serum total cholesterol level, serum high-density lipoprotein level, diabetes status, and smoking history.

**Cardiac CT examination**

Patients with a heart rate >70 bpm were administered beta-blocker (metoprolol) 25–50 mg before scanning to reduce and control their heart rate. The prospective ECG gated axial scanning mode was adopted, and the scanning range was from 1 cm below the trachea forks to the bottom of the heart. The coronary artery calcium score (CACS) was obtained in a single breath hold on a non-contrast CT scan. The contrast agent Visipaque (Iodixanol, 270 mg iodine/ml; GE Healthcare, Ireland) was injected into the median cubital vein at a flow rate of 4.5 to 5.5 ml/s, with an injection dose of 0.6 to 0.8 ml/kg body mass, before rinsing with 30 ml of normal saline. The CCTA scanning parameters were as follows: automatic adjustment of tube current; tube voltage, 120 kVp; rack speed, 0.27 RPM; collimation width, 64 × 0.625 mm; reconstruction layer thickness, 0.9 mm; layer spacing, 0.45 mm; and tube current automatic exposure control index, 13.

**Coronary plaque analysis**

AI Coronary artery CTA (United Imaging, Shanghai, China) was used for quantitative analysis. CACSs were quantified using the Agatston method and divided into 0, 1–99, 100–399, and ≥400, indicating no calcification, mild calcification, moderate calcification, and severe calcification, respectively.

Based on the CCTA images, the CT vulnerable signs of high-risk plaques were accurately identified, including the following four signs: (1) positive remodeling, that is, the ratio of the maximum vessel diameter at the plaque to the normal mean vessel diameter at the proximal and distal end of the plaque was ≥1.1; (2) low-density plaques, that is, plaques with any component whose CT value is less than 30 HU; (3) punctate calcification, that is, calcification in plaques with a CT value >130 HU and diameter <3 mm; and (4) the “napkin ring” sign, that is, the edge of the low-density patch is surrounded by a slightly high-density shadow. A plaque with at least two of these signs at the same time is defined as high-risk. Assessment of plaque properties was performed by two senior cardiac-imaging physicians (each with ≥ 5 years of experience) who were blinded to clinical data. Whenever their readings disagreed, a third physician with > 10 years of experience convened a consensus meeting and made the final decision.

**Abdominal CT scan and VAT analysis**

The scanning parameters of unenhanced abdominal CT imaging were as follows: tube voltage, 120 kVp; automatic tube current modulation technology (dose index＝22, reference value: 162 mAs); rotational speed, 0.75 s; layer spacing, 1.172:1; and layer thickness, 3 mm.

The spectral iterative reconstruction algorithm (Level-3) was used to reconstruct the original data and generate a spectral base image dataset, which was then transferred to a Philips dedicated workstation (IntelliSpace Portal Version 6.5, Philips Healthcare). The obtained spectral base images encompassed conventional polychromatic imaging at 120-kVp, as well as an array of spectral-reconstructed virtual monochromatic imaging (VMI) at various energy levels. The image was automatically segmented on a single axial section at the level of the lumbar 3 vertebrae to distinguish visceral and subcutaneous adipose tissue. The boundary was manually sketched, and the density threshold was set between -190 and -30 Hounsfield Units (HU). The software automatically calculated the VAT area (cm^2^) in this layer. At the same time, the image contrast float bar is adjusted to adjust the photon energy, and the photon energy is increased from 40 keV to 70 keV in 10 keV units to obtain pseudo-color maps. The mean attenuation values of VAT on conventional 120-kVp images, 40-keV VMIs and 70-keV VMIs were defined as CT_poly_, CT_40keV_ and CT_70keV_, respectively. The slope of the spectral curve was calculated as follows: λ_HU_ = (CT_40keV_ - CT_70keV_)/30. Eff-Z was also obtained from the atomic number imaging pseudo-color map.

DECT data were independently measured and analyzed by two experienced cardiovascular radiologists who were blinded to all clinical information and plaque characteristics. The first radiologist randomly selected 60 patients for a second measurement after an interval of 2 weeks and compared the data using intraclass correlation coefficient (ICC) to evaluate the intra-observer consistency (1,1). The inter-observer consistency was assessed by comparing the second radiologist’s measurements with the first radiologist’s measurements (2,1). The above VAT multi-modal indicators show good measurement consistency (ICC >0.80 for all). Finally, the average of the measurements of the two observers was included in the statistical analysis.

**References**

1 Thygesen K, Alpert JS, Jaffe AS et al (2018) Fourth Universal Definition of Myocardial Infarction (2018). Circulation 138:e618-e651

**Supplemental** **Table S1**

Multivariable logistic models for CT_40keV_ derived from DECT as a predictor of high-risk plaque

|  | **OR** | **95% CI** | ***p* value** |
| --- | --- | --- | --- |
| Model 1 | 0.885 | 0.831-0.943 | <0.001* |
| Model 2 | 0.885 | 0.830-0.944 | <0.001* |
| Model 3 | 0.860 | 0.798-0.928 | <0.001* |
| Model 4 | 0.849 | 0.783-0.921 | <0.001* |
| Model 5 | 0.829 | 0.755-0.910 | <0.001* |
| Model 6 | 0.843 | 0.776-0.917 | <0.001* |
| Model 7 | 0.840 | 0.769-0.918 | <0.001* |

DECT: dual-energy computed tomography; OR, odds ratio; CI, conﬁdence interval.

Model 1 was adjusted for age and gender;

Model 2 was adjusted for all covariates in Model 1 plus body mass index;

Model 3 was adjusted for all covariates in Model 2 plus visceral fat area;

Model 4 was adjusted for all covariates in Model 3 plus traditional cardiovascular risk factors (hypertension, diabetes, smoking, drinking, total cholesterol, triglycerides, high-density lipoprotein, low-density lipoprotein);

Model 5 was adjusted for all covariates in Model 4 plus coronary stenosis severity;

Model 6 was adjusted for all covariates in Model 4 plus coronary artery calcium score (CACS);

Model 7 was adjusted for all covariates in Model 4 plus segment involvement score (SIS).

*Indicates *p* ＜0.05.
